# Metabolic and genetic factors affecting the productivity of pyrimidine nucleoside in *Bacillus subtilis*

**DOI:** 10.1186/s12934-015-0237-1

**Published:** 2015-04-15

**Authors:** Hui Zhu, Shao-Mei Yang, Zhao-Min Yuan, Rui Ban

**Affiliations:** Department of Biochemical Engineering, School of Chemical Engineering and Technology, Tianjin University, Tianjin, 300072 China; Key Laboratory of Systems Bioengineering, Ministry of Education, Tianjin University, Tianjin, 300072 China

**Keywords:** *Bacillus subtilis*, Cytidine, Uridine, Gene deletion, Gene expression, Pyrimidine nucleotide biosynthesis

## Abstract

**Background:**

Cytidine and uridine are produced commercially by *Bacillus subtilis.* The production strains of cytidine and uridine were both derivatives from mutagenesis. However, the exact metabolic and genetic factors affecting the productivity remain unknown. Genetic engineering may be a promising approach to identify and confirm these factors.

**Results:**

With the deletion of the *cdd* and *hom* genes, and the deregulation of the *pyr* operon in *Bacillus subtilis*168, the engineered strain produced 200.9 mg/L cytidine, 14.9 mg/L uridine and 960.1 mg/L uracil. Then, the overexpressed *prs* gene led to a dramatic increase of uridine by 25.9 times along with a modest increase of cytidine. Furthermore, the overexpressed *pyrG* gene improved the production of cytidine, uridine and uracil by 259.5%, 11.2% and 68.8%, respectively. Moreover, the overexpression of the *pyrH* gene increasesd the yield of cytidine by 40%, along with a modest augments of uridine and uracil. Lastly, the deletion of the *nupC-pdp* gene resulted in a doubled production of uridine up to 1684.6 mg/L, a 14.4% increase of cytidine to 1423 mg/L, and a 99% decrease of uracil to only 14.2 mg/L.

**Conclusions:**

The deregulation of the *pyr* operon and the overexpression of the *prs*, *pyrG* and *pyrH* genes all contribute to the accumulation of pyrimidine nucleoside compounds in the medium. Among these factors, the overexpression of the *pyrG* and *pyrH* genes can particularly facilitate the production of cytidine. Meanwhile, the deletion of the *nupC-pdp* gene can obviously reduce the production of uracil and simultaneously improve the production of uridine.

**Electronic supplementary material:**

The online version of this article (doi:10.1186/s12934-015-0237-1) contains supplementary material, which is available to authorized users.

## Introduction

**Figure 1:**
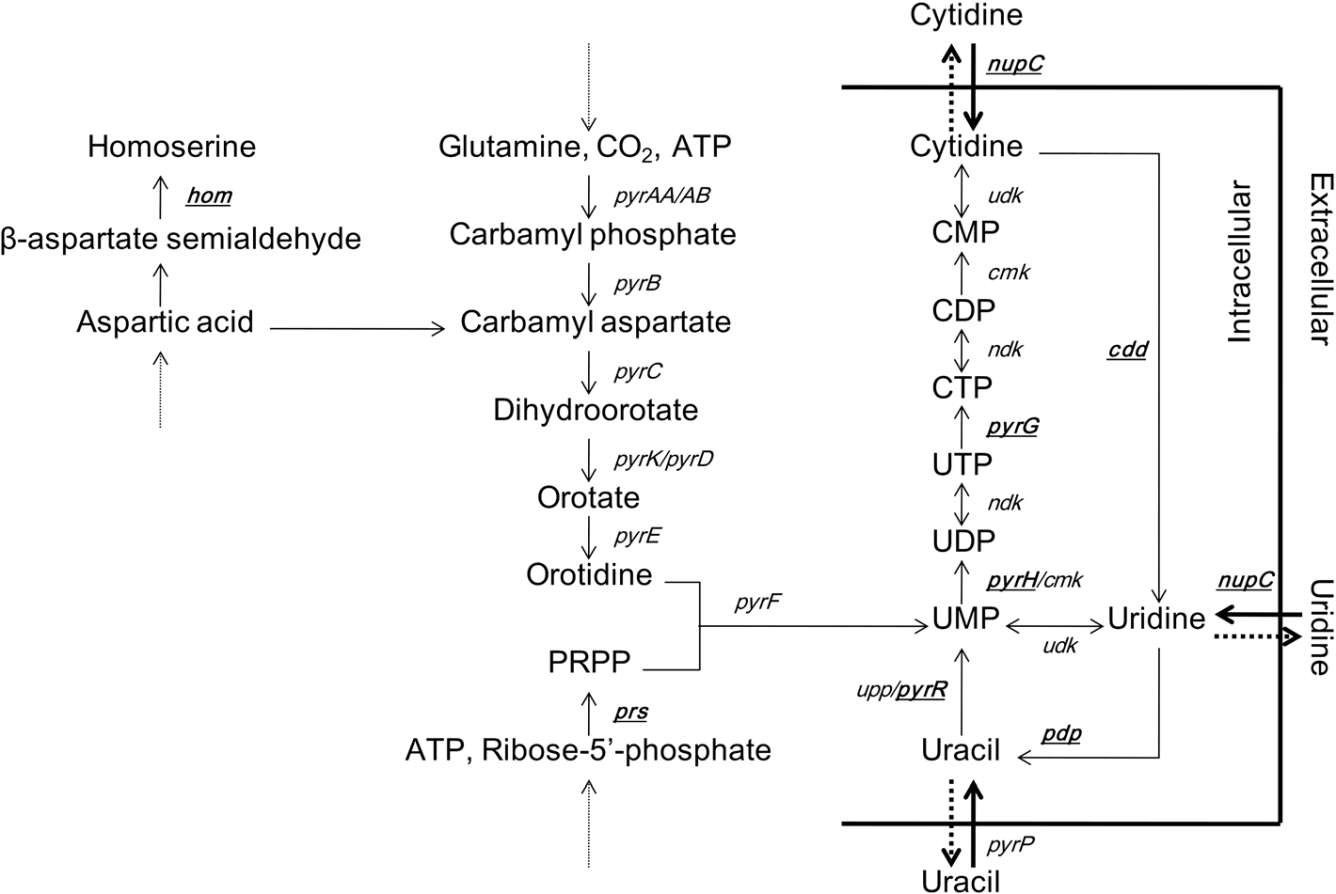
The biosynthetic pathway of pyrimidine nucleotide in *B. subtilis.*

*Bacillus subtilis* is able to synthesize the uridine 5'-monophosphate (UMP) *de novo*. The excess UMP can be further converted to terminal metabolites (cytidine, uridine and uracil), which could then be secreted out of the cell (Figure 1). The pyrimidine nucleotide biosynthetic (*pyr*) operon of *B. subtilis* contains 10 genes. The first gene of the *pyr* operon encodes a bifunctional protein PyrR which is the regulator protein for *pyr* operon and a uracil phosphoribosyl transferase [1]. The second gene, *pyrP*, encodes a uracil permease. The remaining eight genes encode the six enzymes involved in the *de novo* biosynthesis of UMP [2]. The expression of the *pyr* operon is regulated by transcriptional attenuation mechanism involving PyrR. The PyrR protein is mainly activated by UMP and the regulating elements are three specific binding loops (BL1, BL2 and BL3) on the nascent *pyr* mRNA. The combination of the PyrR protein and BLs disrupts the antiterminator, permitting the formation of terminator hairpin and leading to the reduced expression of the downstream genes [3,4]. The resulting high intracellular concentration of UMP would strongly inhibit the transcription of the *pyr* operon.

In *B. subtilis*, L-aspartate is the precursor of both amino acids (lysine, methionine, threonine, and isoleucine) and pyrimidine nucleotide biosynthesis (Figure 1). The deficiency of homoserine dehydrogenase (encoded by the *hom* gene) can prevent the aspartate entering to the methionine and threonine biosynthesis and improve the supplement to the *de novo* biosynthesis of UMP [5]. Phosphoribosyl pyrophosphate (PRPP) is another important precursor of pyrimidine nucleotide biosynthesis (Figure 1). Phosphoribosyl pyrophosphate synthetase (PRS) is encoded by the *prs* gene of the *gcaD-prs-ctc* operon whose transcription regulation mechanism has been unknown yet [6]. Improving the expression level of the *prs* gene can significantly increase the intracellular pool of PRPP [7,8].

In *B. subtilis*, UMP kinase, which is encoded by the *pyrH* gene, catalyzes the phosphorylation of UMP by ATP to yield UDP and ADP (Figure 1). The UMP kinase activity is regulated allosterically by GTP (activator) and UTP (inhibitor) [9,10]. The *pyrG* gene encodes CTP synthetase which aminates UTP to form CTP (Figure 1). The transcription of the *pyrG* gene is tightly regulated by a CTP-sensitive reiterative transcription attenuation control mechanism [11-13]. The inherent regulation of the *pyrG* gene transcription and CTP synthetase activity limit the excessive synthesis of CTP in *B. subtilis.*

Catalyzed by non-specific 5′-phosphatase, the excessive intracellular CMP and UMP can be dephosphorylated to form cytidine and uridine, respectively. The cytidine can be further deaminized to form uridine by cytidine deaminase (encoded by the *cdd* gene) (Figure 1) [14]. In the *dra-nupC-pdp* operon, the *pdp* gene encodes pyrimidine nucleoside phosphorylase which catalyzes the degradation of uridine to form uracil and D-ribose-1-phosphate [15]. Then, uracil will be secreted out of the cell. Therefore, the terminal metabolites of pyrimidine nucleotide are generally uracil instead of cytidine and uridine in the wild-type *B. subtilis.* The *dra* gene encodes deoxyriboaldolase and the *nupC* gene encodes the transporter responsible for pyrimidine nucleoside uptake. The expression of the *dra-nupC-pdp* operon is repressed by glucose and induced by deoxyribonucleosides and deoxyribose [16,17]. When the carbon source is poor, by the physiological function of the *dra-nupC-pdp* operon, the pyrimidine nucleoside accumulated in the medium could be recycled as carbon source. Obviously, the physiological functions of *nupC* and *pdp* genes make no contribution to the accumulation of pyrimidine nucleoside in the medium.

*B. subtilis* strains with defect and pyrimidine analogue resistant could accumulate large amounts of cytidine or uridine [18-21]. Nevertheless, the exact genetic and metabolic mechanisms resulting in pyrimidine nucleoside over-production have not been fully identified and confirmed.

In this study, by using genetic manipulation method, we modified some key genes and operons related to the pyrimidine nucleotide biosynthesis in *B. subtilis* 168 and investigated the influence of these modifications on the production of pyrimidine nucleoside compounds.

## Results

### Deletion of the *cdd* and *hom* genes

**Figure 2:**
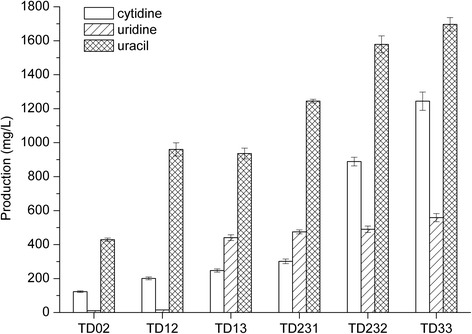
Pyrimidine and pyrimidine nucleoside accumulation by *B. subtilis* strains after 72 h fermentation. Results are the average of three replicates with error bars indicating standard error from the mean (TD02, TD12, TD13, TD231, TD232 and TD33).

In order to observe the effects of the related genetic modification on cytidine and uridine synthesis separately, we blocked the reaction from cytidine to uridine by deleting 151 bp coding sequences of the *cdd* gene in *B. subtilis* 168 N and obtained the *cdd* gene deficient strain *B. subtilis* TD01 (Additional file 1: Figure S1). Subsequently, in order to improve the supplement of aspartate for the pyrimidine nucleotide biosynthesis, we deleted 827 bp coding sequences of the *hom* gene in the strain TD01 and obtained the strain *B. subtilis* TD02 (Additional file 1: Figure S2). The shake-flask culture experiments demonstrated that the strain TD02 could accumulate cytidine and uracil in detectable level in medium while no uridine was detected (Figure 2).

### Deregulation of the *pyr* operon

**Figure 3:**
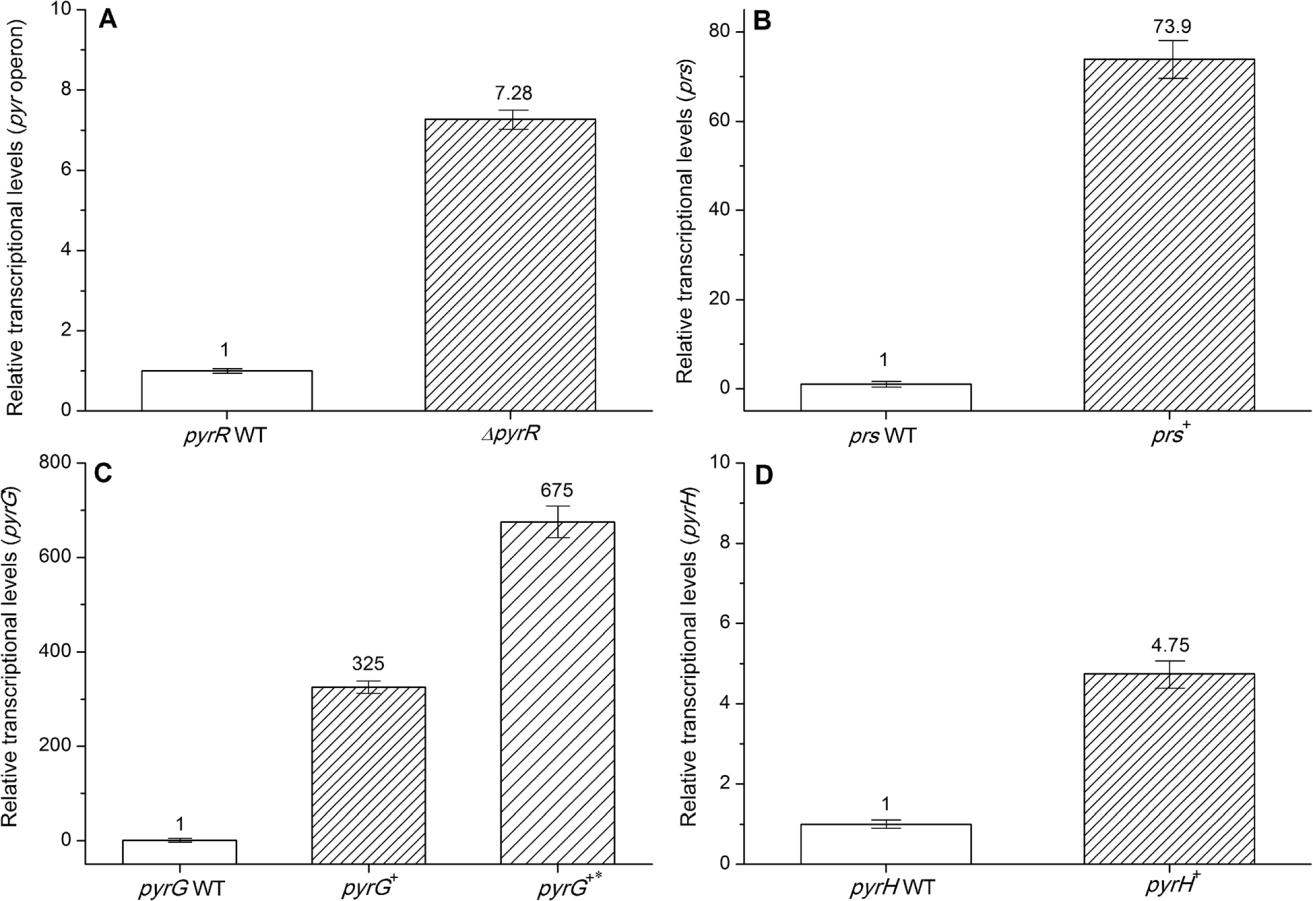
Relative transcriptional levels of *mRNA* in the recombinant strains. The mRNA expression levels of WT strain is regarded as 1(blank). The relative mRNA expression levels of recombinant strains (filled) are compared with WT strain. (A) Relative transcriptional levels of the pyrR gene. *ΔpyrR*: the *pyrR* gene was deleted. (B) Relative transcriptional levels of the prs gene. *prs* ^*+*^: the *prs* gene was overexpressed. (C) Relative transcriptional levels of the pyrG gene. *pyrG* ^*+*^: the *pyrG* gene was constitutively expressed. *pyrG* ^+*^: the *pyrG* gene was overexpressed. (D) Relative transcriptional levels of the pyrH gene. *pyrH* ^*+*^: the *pyrH* gene was overexpressed. The *ccpA* gene was used as the internal control gene to normalize the results. Results are the average of three replications with error bars indicating standard error from the mean.

As described before, the transcription regulation mechanism of the *pyr* operon restricted the over-synthesis of UMP and its derivatives. We deleted 738 bp coding sequences of the *pyrR* gene in the strain TD02 and constructed the recombinant *B. subtilis* TD12 (Additional file 1: Figure S3). The *pyr* operon mRNA transcription level in the *ΔpyrR* strain TD12 was compared with parent strain TD02 through RT-qPCR analysis. We chose the sequences which lay in the middle of the *pyr* operon as the detective point. The transcript abundance increased 6.28-fold in TD12 (Figure 3), which indicated that the *pyr* operon was deregulated in *ΔpyrR* strain. The shake-flask culture experiments showed that the strain TD12 could accumulate 200.9 ± 8.3 mg/L cytidine, 14.9 ± 0.8 mg/L uridine and 960.1 ± 39.1 mg/L uracil, respectively (Figure 2). The deregulation of the *pyr* operon expression could significantly increase the accumulation of both cytidine and uracil, and have little effect on the accumulation of uridine in the medium.

### Overexpression of the *prs* gene

PRPP is not only a precursor for UMP *de novo* biosynthesis but also locates in the crossing point in multiple metabolic pathways. To illustrate the impact of intracellular PRPP level on the biosynthesis of UMP and its derivatives, we overexpressed the *prs* gene in TD12 and constructed recombinant strain *B. subtilis* TD13. The RT-qPCR analyses showed that a 72.86-fold improvement of the *prs* mRNA transcription level occurred in recombinant TD13 compared with parental strain TD12, which indicated that the *prs* gene was successful overexpressed (Figure 3). The flask culture revealed that the recombinant strain TD13 accumulated 247.2 ± 9.6 mg/L cytidine, 440.7 ± 17.1 mg/L uridine and 935.7 ± 31.9 mg/L uricil in medium, respectively (Figure 2). Compared with strain TD12, the accumulation of cytidine and uracil of strain TD13 slightly rose or dropped, but the accumulation of uridine significantly increased by 25.9-fold. These results demonstrated that pyrimidine nucleotide precursor can be further increased by overexpressing the *prs* gene, and intracellular PRPP level was an important rate-limiting factor in biosynthesis of UMP and its derivatives. In other words, the improvement of PRPP supplement could increase the synthesis of pyrimidine nucleoside which was mainly reflected in the improvement of uridine.

### Overexpression of the *pyrG* gene

**Figure 4:**
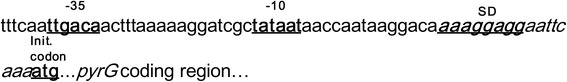
Nucleotide sequence of P_SB_ expression cassette. The sequence of the nontemplate strand is shown. The −10 and −35 regions of the promoter, Shine–Dalgarno sequence (SD) and initiation codon are bold and underlined. The RNA-stabilizing elements are shown in italics.

The CTP synthetase (encoded by the *pyrG* gene) catalyzes the reaction from UTP to CTP. In order to investigate the effects of CTP synthetase level on pyrimidine nucleoside biosynthesis, we constructed the recombinant *B. subtilis* TD231 by introducing 4 extra G residues at the 5′ ends of the *pyrG* transcripts based on the strain TD13 [13]. Meanwhile, on the basis of strain TD13, we constructed recombinant *B. subtilis* TD232 by repleacing the ITR of the *pyrG* gene with the *gsiB* mRNA stabilizer and made the promoter of the *pyrG* gene consensus (Figure 4). The RT-qPCR analysis showed that the intracellular *pyrG* mRNA level of recombinant TD231 and TD232 were about 325-fold and 675-fold, respectively, in comparison to the parent strain TD13 containing the *pyrG* gene (Figure 3). In the flask fermentation medium, the TD231 and TD232 could accumulate 301.3 ± 13.7 mg/L and 888.7 ± 25.7 mg/L cytidine, which achieved a 21.9% and a 259.5% improvement compared to the *pyrG* gene WT strain TD13, respectively (Figure 2). Meanwhile, the uracil production increased about 33% (1244.9 ± 10.9 mg/L) and 68.8% (1579 ± 49.8 mg/L), respectively, while the uridine production only increased ~7.9% (474.6 ± 11.8 mg/L) and ~11.2% (490.1 ± 19.1 mg/L), respectively (Figure 2). These results revealed that the improvement of CTP synthetase level could not only contribute to the production of the downstream metabolites of CTP (known as cytidine), but also those of UMP (known as uracil & uridine). This may be explained by that the improvement of metabolic flux to cytidine led to the reduction of the intracellular UMP pool and subsequently the UMP synthesis was released from UMP-sensitive feedback inhibition to some extent, so that UMP synthesis increased in actually and a part of the increased UMP contributed to the elevation of uridine/uracil production.

### Overexpression of the *pyrH* gene

Among the reactions from UMP to cytidine, UMP kinase (encoded by the *pyrH* gene) might serve as a rate-limiting factor. In order to confirm this, we overexpressed the *pyrH* gene in strain TD232 and obtained the recombinant *B. subtilis* TD33. The RT-qPCR analyses showed that the *pyrH* mRNA level of TD33 was about 3.75-fold higher than that of strain TD232 of which the *pyrH* gene was wild-type (Figure 3). The shake flask fermentation experiments showed that the recombinant TD33 accumulated 1244.2 ± 53.9 mg/L cytidine, 558.6 ± 24.2 mg/L uridine and 1696.7 ± 39.7 mg/L uracil in the culture broth and increased by 40%, 14% and 7.5%, respectively, compared with strain TD232 (Figure 2). The production of cytidine, uridine and uracil all increased with the improvement of UMP kinase express level. These results proved that UMP kinase express level was also a rate-determining factor of pyrimidine nucleoside compounds production.

### Disruption of the *nupC-pdp* gene

To ascertain the effect of the *nupC-pdp* gene on pyrimidine nucleoside accumulation in *B. subtilis*, the *nupC-pdp* gene was deleted from the chromosome of strains TD12, TD13 and TD33, respectively (Additional file 1: Figure S4). The corresponding derivative strains were named as *B. subtilis* TD12np, TD13np and TD33np, respectively. The culture broth analyses of the six strains were shown in Table 1. In the strains (TD13np and TD33np) with the *prs* gene overexpressed, the knockout of the *nupC-pdp* gene led to a decrease of the uracil yield by 99% on average, while an increase of cytidine and uridine yield by 23% and 230% on average, respectively. In strain TD12 (the *prs* gene is wild-type), the knockout of the *nupC-pdp* gene led to a decreased accumulation of uracil by about 50%, while an increased accumulation of cytidine and uridine by about 0.3-fold and 79-fold, respectively. These results indicated that the accumulated uracil in medium was mainly derived from the degradation of uridine catalyzed by pyrimidine nucleoside phosphorylase. Meanwhile, there may be another metabolic reaction remains unknown, which could generate uracil in *B. subtlis*, and the rate of the unknown reaction may be controlled by intracellular PRPP pool level.

**Table 1:** **Pyrimidine and pyrimidine nucleoside produced by modifying different genes of the pyrimidine nucleotide biosynthesis pathway in** ***B. subtilis*** **after 72 h fermentation**

**Strain**	**Productivity (mg/L)**	**Ratio (%)**	**Productivity (mg/L)**	**Ratio (%)**	**Productivity (mg/L)**	**Ratio (%)**	**Molarity (mmol/L)**
	**Cytidine**		**Uridine**		**Uracil**		**Total**
TD12	200.9 ± 8.3		14.9 ± 0.8		960.1 ± 39.1		9.5
TD12np	260.3 ± 11.3	129.6	1187.5 ± 49.9	7970	508.2 ± 18.5	52.9	10.5
TD13	247.2 ± 9.6		440.7 ± 17.1		935.7 ± 31.9		11.2
TD13np	326.9 ± 7.0	132.2	1571.4 ± 38.9	356.6	16.9 ± 0.1	1.8	7.9
TD33	1244.2 ± 53.9		558.6 ± 24.2		1696.7 ± 39.6		22.6
TD33np	1423.0 ± 47.5	114.4	1684.6 ± 62.4	301.6	14.2 ± 0.2	0.8	22.9

Data shown are the average of three independent experiments.

Together with the inactivation of the *cdd*, *hom* and *pyrR* genes, the deletion of *nupC-pdp* gene led to a slight increase of the total amount of pyrimidine compounds (i.e., cytidine, uridine, and uracil) accumulated in the culture. However, together with the inactivation of the *cdd*, *hom* and *pyrR* genes and the overexpression of the *prs* gene, the deletion of the *nupC-pdp* gene led to a modest decrease of the total amount of pyrimidine compounds in the culture. Nevertheless, if the *pyrH* and *pyrG* genes were both overexpressed subsequently, the total amount of pyrimidine compounds in the culture would be doubled to about 22 mmol/L (Table 1). These results revealed that the overexpression of the *pyrH* and *pyrG* genes caused a metabolic flux enlargement from UMP to cytidine, thereby, reducing the intracellular UMP level which may relieve feedback inhibition for UMP to carbamyl phosphate synthetase and lead to more UMP synthesis.

### The growth of recombinant strains

**Figure 5:**
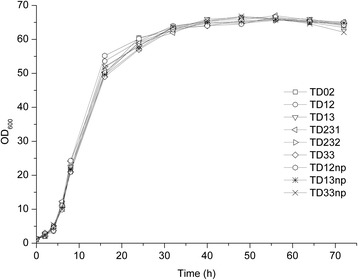
The biomass of recombinant strains in 72 h fermentation. Data obtained are the result of three independent fermentations.

In shaking flask fermentation, we measured the biomass of the recombinant strains mentioned above. The data showed that the cell growth of recombinant strains were similar to that of the parental strains (Figure 5). Therefore, the above modification of relevant genes of pyrimidine nucleotide biosynthesis had no detectable effect on cell growth.

## Discussion

As already shown in the “introduction”, the pyrimidine nucleotide biosynthesis of *B. subtilis* was strictly regulated so that no excess pyrimidine nucleoside would be synthesized and secreted to the medium. In order to well illuminate the rate-determining factors affecting pyrimidine nucleoside excess synthesis, we chose genetic engineering as a desired strategy.

The inactivation of the *cdd* gene abolished the reaction from cytidine to uridine and resulted in the accumulation of cytidine in the medium, indicating that the deficiency of the *cdd* gene is a key factor of the accumulation of cytidine. The result coincided with the experimental findings of previous studies [22].

Based on the *hom* and *cdd* genes deficiency, the deregulation of the *pyr* operon by deleting the *pyrR* gene doubled the yield of both cytidine and uracil, while mutagenesis was the mere prevailing method to achieve the same genetic effect in the past [21]. However, though one determining factor of UMP biosynthesis have been revealed with deregulation of the *pyr* operon, the carbamoyl phosphate synthetase (encoded by the *pyrAA*/*pyrAB* gene), which catalyzes the first reaction of UMP *de novo* biosynthesis, is still subjected to feedback inhibition by UMP [23,24]. Hence, the excess synthesis of pyrimidine nucleoside compounds is still considered to be limited.

The overexpression of the *prs* gene resulted in an increase of uridine yield by 25.9-fold in this work. Analogous experimental results also existed in the study of biosynthesis of purine nucleoside and its derivatives in *B. subtilis* [8,25]. Thus, PRPP pool level is one important restrictive factor affecting the overproduction of nucleotide and its derivatives. However, PRS is an allosteric enzyme and its activity is intensively feedback inhibited by purine nucleotide [26]. By using site-directed mutation to release the feedback inhibition of phosphoribosyl pyrophosphate synthetase (PRS), satisfactory results may be obtained.

The overexpression of the *pyrG* and *pyrH* genes resulted in a significantly increased amount of cytidine in the medium, proving that the activity of both UMP kinase and CTP synthetase were rate-determining factors for cytidine over-synthesis. Since both UMP kinase and CTP synthetase are allosteric enzymes, it is believed that by releasing their feedback inhibition, the cytidine production could be further improved. The overexpression of the *pyrG* and *pyrH* genes also led to an increase in the total pyrimidine compounds accumulation in the medium. We interpret that the augmented metabolic flux from UMP to CTP could reduce the intracellular UMP level and thereby release the feedback inhibition on UMP biosynthesis, especially the activity of carbamyl phosphate synthetase.

The pyrimidine nucleoside phosphorylase (encoded by the *pdp* gene) involves in the reaction from uridine to uracil and the loss of the *pdp* gene should make cell accumulate only nucleosides and no uracil in theory [27,28]. However, according to the experimental results, by deleting the *nupC-pdp* gene, we obtained that the uracil accumulation reduced by 99% rather than 100%. The small amount of uracil remaining in the medium likely resulted from some unspecific reactions. Since the decrease of uracil yield was always accompanied with a remarkable increase of uridine yield, the uracil accumulated in the medium was mainly derived from uridine or UMP. Under the precondition of the *pdp* gene deficiency, the amount of residual uracil in the medium seems to be negatively correlated with the amount of the available PRPP in the cell (TD12np). Hence, the unspecific reactions which result in the residual uracil are likely subject to the level of intercellular PRPP pool.

## Conclusions

We deleted/overexpressed the genes which were closely related to pyrimidine nucleoside biosynthesis by using genetic manipulation method in *B. subtilis*168 and constructed a series of recombinant strains. The results of shaking flask fermentation demonstrated that the deregulation of *pyr* operon, the overexpression of the *prs*, *pyrG* and *pyrH* genes, and the deletion of the *nupC-pdp* gene all facilitated the over-synthesis of pyrimidine nucleoside compounds. The production of uridine and cytidine were up to 1684.6 mg/L and 1423 mg/L in the result strain TD33np, respectively. (i) The UMP synthesis operon (*pyr* operon) and the PRPP synthesis (encoded by the *prs* gene) are both rate-determining factors for the UMP biosynthesis. If the feedback inhibitions of PRPP synthetase and carbamoyl phosphate synthetaserelease are released, the production of pyrimidine nucleoside compounds may be improved further. (ii) The overexpression of the *pyrH* and *pyrG* genes can improve the proportion of cytidine in the pyrimidine nucleoside products, while reduce the proportion of uridine. If the feedback inhibition of the CTP synthetase and UMP kinase are released, the proportion of cytidine in the pyrimidine nucleoside products may be improved further. (iii) The pyrimidine nucleoside phosphorylase (encoded by the *pdp* gene) activity is of close correlation with the accumulation of uracil in the medium, and the deletion of the *nupC-pdp* gene can reduce the accumulation of uracil to a very low level (1%) in the medium. (iv) The *cdd* gene is a key factor of the accumulation of cytidine, and if cytidine deaminase activity is restored, the proportion of uridine in the accumulated pyrimidine compounds will increase sharply.

## Materials and methods

### The bacterial strains and general culture conditions

The strains used in this study are listed in Table 2. All organisms were cultured in Luria-Bertani (LB) medium or on 1.5% (w/v) agar plates supplemented with the appropriate antibiotics when required. Chloramphenicols (Cm), 6 μg ml^−1^and neomycin (Nm), 15 μg ml^−1^, were used to select resistant *B. subtilis* cells. For liquid cultures, *B. subtilis* were incubated at 37°C and were shaken at 220 rpm.

**Table 2:** **Strains**

**Strain**	**Relevant genotype**	**Reference**
*B.subtilis* 168 (BGSC 1A1)	*trpC2*	Laboratory stock
*B.subtilis* 168 N	*trpC2, ΔaraR::neo* ^*R*^	Laboratory stock
*B.subtilis* TD01	*trpC2, ΔaraR::neo* ^*R*^ *,Δcdd*	This study
*B.subtilis* TD02	*trpC2, ΔaraR::neo* ^*R*^ *, Δcdd, Δhom*	This study
*B.subtilis* TD12	*trpC2,ΔaraR::neo* ^*R*^ *, Δcdd, Δhom, ΔpyrR*	This study
*B.subtilis* TD12np	*trpC2,ΔaraR::neo* ^*R*^ *, Δcdd, Δhom, ΔpyrR, ΔnupC-pdp*	This study
*B.subtilis* TD13	*trpC2,ΔaraR::neo* ^*R*^ *, Δcdd, Δhom,ΔpyrR, ΔxylR::prs*	This study
*B.subtilis* TD13np	*trpC2,ΔaraR::neo* ^*R*^ *, Δcdd, Δhom,ΔpyrR, ΔxylR::prs, ΔnupC-pdp*	This study
*B.subtilis* TD231	*trpC2,ΔaraR::neo* ^*R*^ *, Δcdd, Δhom, ΔpyrR, ΔxylR::prs, pyrG* ^*+*^	This study
*B.subtilis* TD232	*trpC2,ΔaraR::neo* ^*R*^ *, Δcdd, Δhom, ΔpyrR,ΔxylR::prs, pyrG* ^+*^	This study
*B.subtilis* TD33	*trpC2,ΔaraR::neo* ^*R*^ *, Δcdd, Δhom, ΔpyrR, ΔxylR::prs,pyrG* ^+*^ *,pyrH* ^*+*^	This study
*B.subtilis* TD33np	*trpC2,ΔaraR::neo* ^*R*^ *, Δcdd, Δhom, ΔpyrR,ΔxylR::prs,pyrG* ^+*^ *,pyrH* ^*+*^ *, ΔnupC-pdp*	This study

*pyrG* ^*+*^: the *pyrG* gene was constitutively expressed. *pyrG* ^+*^: the *pyrG* gene was overexpressed.

### DNA manipulation techniques and PCR

The isolation and manipulation of DNA were conducted according to standard procedures [29]. All chromosomal DNA were extracted from *B. subtilis* and isolated by the protocol of Sangon Biotech (Shanghai, China). PCR was performed with DNA Ploymerase HiFi or Taq DNA polymerase (TransGen, Beijing, China) in a DNA thermal cycler (DNAEngine, BIO-RED, Hercules, CA, USA) through the procedure recommended by the manufacturer. Overlapped extension PCR (SOE-PCR) was carried out as decribed [30,31]. PCR products were purified with a PCR Purification kit (Biomed, Beijing, China) and analyzed by electrophoresis in 1% (w/v) agarose gels.

### Transformation and transformants’ seletion

Transformation of *B. subtilis* was performed by using competent cells as described by Anagnostopoulos and Spizizen [32]. Competent transformation used liner DNA frangments. The transformants seletion relied on the method described by Liu et al. [33,34].

### Gene deletion

The method of marker-free gene deletion was as described by Liu et al. [33,34]. We took *pyrR* gene as an example. The fragment using for deleting the *pyrR* gene was constructed as follows. The 0.9 kb *cat* (C) fragment was amplified from the pC194 plasmid using the primers Cat1qr and Cat2r (Table 3) [35]. The 1.2 kb araR (R) fragment, including the whole coding region of the *araR* gene, was amplified from the *B. subtilis* 168 genome using the primers araR1qr and araR2qr. The 1.3 kb UPpyrR (U), 0.9 kb DNpyrR (D) and 0.5 kb GpyrR (G) fragments were amplified from the *B. subtilis* 168 genome using the primers pyrUP1 and pyrUP2, pyrDN1q and pyrDN2, and pyrG1and pyrG2, respectively. These five PCR fragments were then ligated in the order U-D-C-R-G by splicing by overlapped extension PCR (SOE-PCR) using the primers pyrUP1 and pyrG2 and then were used to transform. The deletions of the *cdd*, *hom* and *nupC-pdp* genes were similar to the *pyrR* gene.

**Table 3:** **Primers and synthetized fragments**

**Oligonucleotides for** ***pyrR*** **gene deletion**	
Primer number	Sequence 5'- 3'
pyrUP1	AAAAGTGAGCGGATTGA
pyrUP2	TCCTGCCAGAGCATAGAG
pyrDN1q	CTCTATGCTCTGGCAGGAGGGGTTTTTTCTTCAACAATCAGGGGGAAAT
pyrDN2	GGGCCCGGATCCCACTGTCACCCATAATAGAGC
pyrG1	GCATTCTTGTCGGCATTA
pyrG2	GCCACAGCAGGACTCATT
Cat1qr	CATAAAAGCAGGTCTTCATCGCTCTATTATGTCTTCAACTAAAGCACCCAT
Cat2pr	GGGCCCGGATCCTCTTCAACTAAAGCACCCAT
araR1qr	CTGCCCCGTTAGTTGAAGGCATTTTCTGTCAATGTTTTC
araR2qr	AATCCCTCTTGTCTTAATGCTTATTCATTCAGTTTTCGTG
Oligonucleotides for *pyrH* gene overexpression	
Primer number	Sequence 5'- 3'
pyrHUP1	TACGGCATTCACATCAGG
pyrHUP2q	TCCACTTCATCCACTCCATCGCTTAACGCATTGATATGA
P1h	ATGGAGTGGATGAAGTGGA
P2h	CATTCTTTACCCTCTCCTTT
pyrH1q	AAAGGAGAGGGTAAAGAATGGAAAAACCAAAATACAAACG
pyrH2q	CCTTCTACACGATATGTGCTGCGTACAGTAGCCAATTCG
pyrHDN1	AGCACATATCGTGTAGAAGG
pyrHDN2q	ATGGGTGCTTTAGTTGAAGAAATGGCTGTCGCTATTGTT
Cat2h	GCTGTAATATAAAAACCTTC
Cat1qh	AAGAAGAAGGCAATGACACGTCTTCAACTAAAGCACCCAT
araR1qh	CTGCCCCGTTAGTTGAAGGCATTTTCTGTCAATGTTTTC
araR2h	TTATTCATTCAGTTTTCGTG
pyrHG1q	CACGAAAACTGAATGAATAATCAGCCTAATGATGTCTTGT
pyrHG2	ACTTCTTGAACGACTTCCA
Promoter-h	ATGGAGTGGATGAAGTGGAATCGTTTTAGAATGGGAGAATTAACTATTAATGTTTGACAACTATTACAGAGTATGCTATAATAAATTCACAGAATAGTCTTTTAAGTAAGTCTACTCTGAATTTTTTTAAAAGGAGAGGGTAAAGA
CX1h	ATGGAGTGGATGAAGTGGA
CX2h	GCGTACAGTAGCCAATTCG
Oligonucleotides for *prs* gene overexpression	
Primer number	Sequence 5'- 3'
prsUP1	AATCCGCCGCTTCCAA
prsUP2q	TGTCAAACATTAATAGTTAATTCTCCCATTCTAAAACGATTCCACTTCATCCACTCCAT
p1p	CAAATGGAGTGGATGAAG
p2p	ACTTCCCCGTCACTAAAT
prs1	TTAAAAGGAGAGGGTAAAGAATGTCTAATCAATACGGAGATAAG
prs2	TTAGCTGAACAGATAGCTGACT
prsDN1q	AGTCAGCTATCTGTTCAGCTAATGTCCTCCATTGTGATTGAT
prsDN2	ACGCATGATGAAGAACTTG
CR1qp	CCAAGTTCTTCATCATGCGTTCTTCAACTAAAGCACCCAT
CR2p	TTATTCATTCAGTTTTCGTG
prsG1q	CACGAAAACTGAATGAATAAATCAAGTGGCGGAAGAAG
prsG2	AGGATGCGATTCAATTATGC
Promoter-p	CAAATGGAGTGGATGAAGTGGAATCGTTTTAGAATGGGAGAATTAACTATTAATGTTTGACAACTATTACAGAGTATGCTATAATAAATTCACAGAATAGTCTTTTAAGTAAGTCTACTCTGAATTTTTTTAAAAGGAGAGGGTAAAGAATGTCTAATCAATACGGAGATAAGAATTTAAAGATTTTTTCTTTGAATTCGAATCCAGAGCTTGCAAAAGAAATCGCATATATAGTTGGAGTTCAATTAGGGAAATGTTCTGTCACAAGATTTAGTGACGGGGAAGT
CX1p	TCCGCCGATTACTTCTTG
CX2p	CCATGTCACTATTGCTTCAG
Oligonucleotides for *hom* gene deletion	
Primer number	Sequence 5'- 3'
homUP1	TGAACTGACATTTGAACAT
homUP2	CCTTTCTTTTGATTGTCC
homDN1q	CCAAGAGGACAATCAAAAGACGCTTTCTGCTGTTCATAA
homDN2	CGTGTCATTGCCTTCTTC
Cat1qm	AAGAAGAAGGCAATGACACGTCTTCAACTAAAGCACCCAT
Cat2m	GCTGTAATATAAAAACCTTC
araR1qm	CTGCCCCGTTAGTTGAAGGCATTTTCTGTCAATGTTTTC
araR2m	TTATTCATTCAGTTTTCGTG
homG1q	CACGAAAACTGAATGAATAAAAGCGATTCGTGTAGGG
homG2	GATGGTCAGGAAGCAGT
Oligonucleotides for *nupC-pdp gene* deletion	
Primer number	Sequence 5'- 3'
pnUP1	GAAGTGTGCGAAGGATGT
pnUP2	GAGGAGAATGTAGCCAAGAA
pnDN1q	GCAATTTATTCTTGGCTACATTCATTGGACGAAGCGAGAG
pnDN2	CTGCGGAGTTCCTTGTATC
CR1qpn	ATGATACAAGGAACTCCGCAGTCTTCAACTAAAGCACCCAT
CR2pn	TTATTCATTCAGTTTTCGTG
pnG1q	CACGAAAACTGAATGAATAAATCTGCGATGTCAACTGTAT
pnG2	GGCTGCGTCTTCTTCTGTT
Oligonucleotides for *cdd* gene deletion	
Primer number	Sequence 5'- 3'
cddUP1	GCTTCAGGACGGACAGTTCAGT
cddUP2q	AGATACTGGTCCAGGGGTGTCAGCACTTGGATTTGGAATACGGCG
cddDN1	GCTGACACCCCTGGACCAGTATCT
cddDN2q	CGTTTGTTGAACTAATGGGTGCTTTCAGAACCGTTGTCCTGCCCTTT
Cat1c	CACCCATTAGTTCAACAAACG
Cat2c	TTCAACTAACGGGGCAG
araR1qc	CTGCCCCGTTAGTTGAAGGCATTTTCTGTCAATGTTTTC
araR2E	CGC**GGATCC**TGACACCCCTGGACCAGTATCT
cddG1E	CGC**GGATCC**TGACACCCCTGGACCAGTATCT
cddG2	CCCACAATTCAAGGTAGACACG
Oligonucleotides for *pyrG* gene constitutive expression and overexpression	
Primer number	Sequence 5'- 3'
pyrGUP1	GGATACGGCGATGAAGGT
pyrGUP2q	GTTCTCTCTTCGTTTTTGAAGAGCCCCCCAAAATACATACTACATAGTTCGAC
pyrGDN1	TTCAAAAACGAAGAGAGAACATAG
pyrGDN2q	GTTTGTTGAACTAATGGGTGCTAATCGTTTGAGACAGGTTGC
pyrGG1	GTCCGCACGAAAACTGAATGAATACCAGCACGGTGAAGTATT
pyrGG2	CCAAGATCCTCAACATCCTT
Cat1g	CACCCATTAGTTCAACAAACG
Cat2g	TTCAACTAACGGGGCAG
araR1qg	CTGCCCCGTTAGTTGAAGGCATTTTCTGTCAATGTTTTC
araR2g	TTATTCATTCAGTTTTCGTG
pyrGUP1b	TACGGCGATGAAGGTAAC
pyrGUP2qb	AGCTCCCTTTCAATTTCTTGCGTACTATGTTCTCTCTTCGT
gsiB1	CAAGAAATTGAAAGGGAGCT
gsiB2	TTCCAAGTGAGGATACAACTCC
pyrGDCRG1	GGAGTTGTATCCTCACTTGG
pyrGDCRG2	CAATGATGCCGTCTGTTC
Promoter-g	CAAGAAATTGAAAGGGAGCTATGTTTTTCTCAAATTGTAAGTTTATTTCATTGACAACTTTAAAAAGGATCGCTATAATAACCAATAAGGACAAAAGGAGGAATTCAAAATGACGAAATATATTTTTGTAACCGGGGGAGTTGTATCCTCACTTGGAA
CX1g	ATGGAGTGGATGAAGTGGA
CX2g	GCGTACAGTAGCCAATTCG

### Gene overexpression

**Figure 6:**
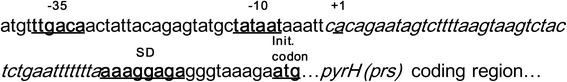
Nucleotide sequence of P_AE_ expression cassette. The sequence of the nontemplate strand is shown. The −10 and −35 regions of the promoter, transcription start site (+1), Shine–Dalgarno sequence (SD) and initiation codon are bold and underlined. The RNA-stabilizing elements are shown in italics.

To overexpress the *pyrH* gene, the inherent ITR of the *pyrH* was replaced by a stronger promoter A1 of bacteriophage *φ*29 ligated to the mRNA stabilizer of the *aprE* gene (P_AE_ expression cassette) (Figure 6). To overexpress the *prs* gene, another *prs* gene copy controlling by P_AE_ expression cassette was integrated in chromosome the *xylR* gene locus. The method to overexpress the *pyrG* gene is that a standardized promoter along with the the *gsiB* mRNA stabilizer (P_SB_ expression cassette) was inserted after the inherent *pyrG* promoter (Figure 4). The method to constitutively express the *pyrG* gene is that 4 extra G residues at the 5′ ends of the *pyrG* ITR. The method of marker-free gene modification was derived from Liu et al. [33,34]. The specific method of gene modification was described as below; for example, the modified the *pyrH* gene fragment was constructed as follows. The 0.9 kb *cat* (C) fragment was amplified from the pC194 plasmid using the primers Cat1qh and Cat2h (Table 3). The 1.2 kb araR (R) fragment was amplified from the *B. subtilis* 168 genome using the primers araR1qh and araR2. The 1 kb UPpyrH (U), 0.7 kb DNpyrH (D) and 0.6 kb GpyrH (G) fragments were amplified from the *B. subtilis* 168 genome using the primers pyrHUP1 and pyrHUP2q, pyrHDN1q and pyrHDN2, and pyrHG1q and pyrHG2, respectively. The 0.8 kb pyrH (H) fragment, including the whole coding region of the *pyrH* gene, was amplified from the *B. subtilis* 168 genome using the primers pyrH1q and pyrH2q. The 0.15 kb Promoter (P) fragment was whole sequence synthesized by AuGCT DNA-SYN Biotechnology Corporation (Beijing, China), including P_AE_ expression cassette, and ligated to plasmid pGH-A0981Gn. P fragment was amplified from plasmid pGH-A0981Gn by using the primers p1h and p2h. These seven PCR fragments were then ligated in the order G-R-C-D-H-P-U by splicing by SOE-PCR using the primers pyrHUP1 and pyrHG2 and then be used to transform. DNA sequencing was done at AuGCT DNA-SYN Biotechnology Corporation (Beijing, China) by using primers CX1h and CX2h. The constitutive expression and overexpression of the *pyrG* gene, and the overexpressions of the *prs* gene were similar to the *pyrH* gene.

### Fermentation

A loop of cells grown on an agar plate of LB medium was inoculated into a 250 mL flask containing 30 mL of stock culture medium (2% glucose, 2% soybean meal hydrolysate, 1% yeast extract, 0.25% NaCl, 0.1% MgSO_4_.7H_2_O, 0.1% KH_2_PO_4_, 0.5% sodiumglutamate, pH = 7.0) and was then cultured for 16 h at 37°C, with shaking at 220 rpm. 1.5 mL of the culture was transferred to a 250 mL flask containing 30 mL of fermentation medium (8% glucose, 3% soybean meal hydrolysate, 2% cornsteepliquor, 1.5% yeast extract, 0.25% NaCl, 0.8% MgSO_4_.7H_2_O, 0.25% KH_2_PO_4_, 1.5% (NH_4_)_2_SO_4,_ 1.5% sodiumglutamate, pH = 7.0) and was then cultured for 72 h at 37°C, with shaking at 220 rpm. Samples were drawn at various time-points during the fermentation and were analyzed for cell growth (OD_600_) and pyrimidine compounds content.

### Fermentation compounds analysis

The qualitative analysis of pyrimidine nucleoside and pyrimidine compounds in the medium was conducted by massspectrograph (Q-Exactive, Thermo Scientific, Waltham, MA, USA).The quantitative analysis of cytidine, uridine and uracil were conducted by HPLC (Waters 2695, Waters, Milford, MA, USA) with an HYPERSIL ODS C18 column (Thermo Scientific, Waltham, MA, USA). Separation was performed at 40°C with 0.05 M KH_2_PO_4_ at a flowrate of 1.5 mL min^−1^. The detective wavelength was 270 nm.

### Quantitative real-time reverse transcription (RT)-PCR analysis

Total RNA of *B. subtilis* was extracted with RNAprep pure Cell/Bacteria Kit (TIANGEN, Beijing, China) as recommended by the supplier and was reverse-transcribed in cDNA using the Maxima First Strand cDNA Synthesis Kit (Thermo Scientific, Waltham, MA, USA) according to the manufacturer’s instructions. The Real-time PCR was carried out by LightCycler 480 (Roche Diagnostics GmbH, Mannheim, Germany) using SYBR Green Ι Master (Roche Diagnostics GmbH, Mannheim, Germany). The *ccpA* gene was served as internal control of Real-time PCR. The quantification cycle (Cq) was determined according to the second derivative maximum method using the LightCycler software 4.1 (Roche Diagnostics GmbH, Mannheim, Germany). The relative expression ratio (RE) was calculated according to Pfaffl [36].

## Additional file

Additional file 1:
**The identification of the gene deletion.** **Figure S1** The identification of the *cdd* gene deletion. (A) Lane 1: marker. Lanes 2–7: PCR products of the recombinants (lanes 2–6, 2004 bp) and the WT strain (lane 7, 2155 bp) by using the primers cddUP1 and cddG2. (B) Lane 1: marker. Lanes 2–7: PCR products of the recombinants (lanes 2–6, 512 bp, 604 bp and 888 bp) and the WT strain (lane 7, 512 bp, 604 bp and 1039 bp) digested by *Sac*I and *Eco*RI. **Figure S2** The identification of the *hom* gene deletion. Lane 1: marker. Lanes 2, 4: PCR products of the WT strain (lane 2, 2531 bp) and the recombinant (lane 4, 1700 bp) by using the primers homUP1 and homDN2. Lanes 3, 5: PCR products of the WT strain (lane 3, 619 bp and 1912 bp) and the recombinant (lane 5, 619 bp and 1081 bp) digested by *Sph*I. **Figure S3** The identification of the *pyrR* gene deletion**.** Lane 1: marker. Lanes 2, 4: PCR products of the WT strain (lane 2, 2642 bp) and the recombinant (lane 4, 1904 bp) by using the primers pyrUP1 and pyrDN2. Lanes 3, 5: PCR products of the WT strain (lane 3, 531 bp and 2111 bp) and the recombinant (lane 5, 531 bp and 1373 bp) digested by *Pst*I. **Figure S4** The identification of the *nupC-pdp* gene deletion. Lane 1: marker. Lanes 2, 5: PCR products of the WT strain (lane 2, 3433 bp) and the recombinant (lane 5, 1495 bp) by using the primers pnUP1 and pnDN2. Lanes 3, 6: PCR products of the WT strain (lane 3, 474 bp and 2959 bp) and the recombinant (lane 6, 474 bp and 1021 bp) digested by *Hin*dIII. Lanes 4, 7: PCR products of the WT strain (lane 4, 174 bp, 339 bp, 1452 bp and 1468 bp) and the recombinant (lane 7, 174 bp, 339 bp and 982 bp) digested by *Eco*RV.
