# Supplementary material for: Metabolic and genetic factors affecting the productivity of pyrimidine nucleoside in Bacillus subtilis
Source: Microb Cell Fact. 2015 Apr 15;14:54. doi: 10.1186/s12934-015-0237-1 (PMC4403831; doi:10.1186/s12934-015-0237-1)
Supplement: Additional file 1: — The identification of the gene deletion. Figure S1 The identification of the cdd gene deletion. (A) Lane 1: marker. Lanes 2–7: PCR products of the recombinants (lanes 2–6, 2004 bp) and the WT strain (lane 7, 2155 bp) by using the primers cddUP1 and cddG2. (B) Lane 1: marker. Lanes 2–7: PCR products of the recombinants (lanes 2–6, 512 bp, 604 bp and 888 bp) and the WT strain (lane 7, 512 bp, 604 bp and 1039 bp) digested by SacI and EcoRI. Figure S2 The identification of the hom gene deletion. Lane 1: marker. Lanes 2, 4: PCR products of the WT strain (lane 2, 2531 bp) and the recombinant (lane 4, 1700 bp) by using the primers homUP1 and homDN2. Lanes 3, 5: PCR products of the WT strain (lane 3, 619 bp and 1912 bp) and the recombinant (lane 5, 619 bp and 1081 bp) digested by SphI. Figure S3 The identification of the pyrR gene deletion. Lane 1: marker. Lanes 2, 4: PCR products of the WT strain (lane 2, 2642 bp) and the recombinant (lane 4, 1904 bp) by using the primers pyrUP1 and pyrDN2. Lanes 3, 5: PCR products of the WT strain (lane 3, 531 bp and 2111 bp) and the recombinant (lane 5, 531 bp and 1373 bp) digested by PstI. Figure S4 The identification of the nupC-pdp gene deletion. Lane 1: marker. Lanes 2, 5: PCR products of the WT strain (lane 2, 3433 bp) and the recombinant (lane 5, 1495 bp) by using the primers pnUP1 and pnDN2. Lanes 3, 6: PCR products of the WT strain (lane 3, 474 bp and 2959 bp) and the recombinant (lane 6, 474 bp and 1021 bp) digested by HindIII. Lanes 4, 7: PCR products of the WT strain (lane 4, 174 bp, 339 bp, 1452 bp and 1468 bp) and the recombinant (lane 7, 174 bp, 339 bp and 982 bp) digested by EcoRV. [file 12934_2015_237_MOESM1_ESM.docx]

**Supplementary material 1**


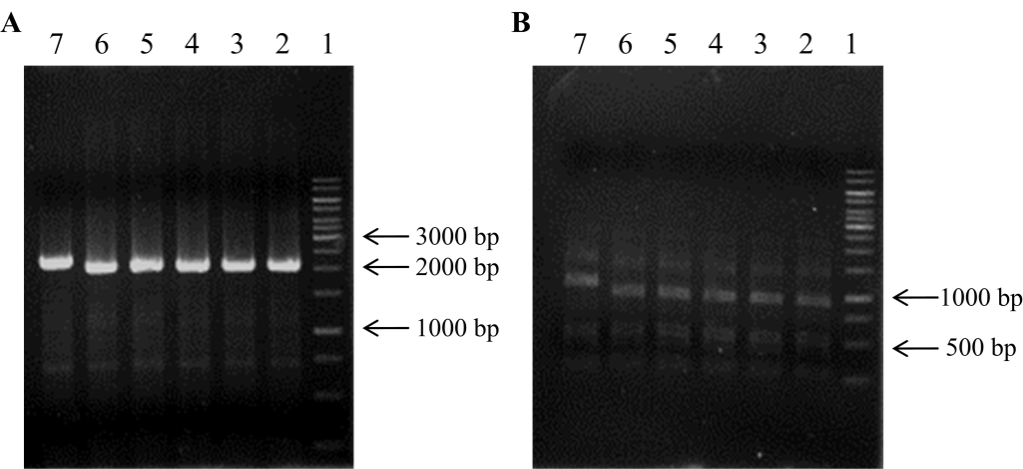


**Figure S1 The identification of the *cdd* gene deletion.** (A) Lane 1: marker. Lanes 2-7: PCR products of the recombinants (lanes 2-6, 2004 bp) and the WT strain (lane 7, 2155 bp) by using the primers cddUP1 and cddG2. (B) Lane 1: marker. Lanes 2-7: PCR products of the recombinants (lanes 2-6, 512 bp, 604 bp and 888 bp) and the WT strain (lane 7, 512 bp, 604 bp and 1039 bp) digested by *Sac*Ⅰ and *Eco*RⅠ.


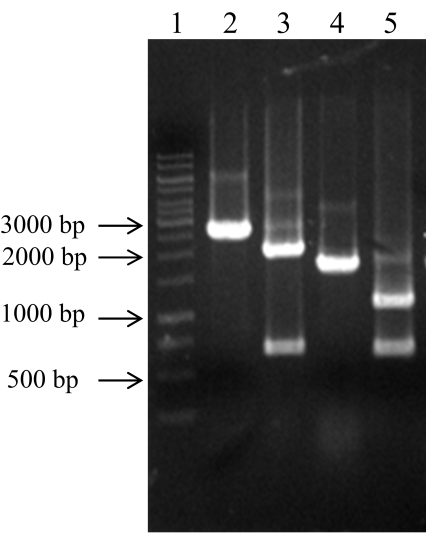


**Figure S2 The identification of the *hom* gene deletion.** Lane 1: marker. Lanes 2, 4: PCR products of the WT strain (lane 2, 2531 bp) and the recombinant (lane 4, 1700 bp) by using the primers homUP1 and homDN2. Lanes 3, 5: PCR products of the WT strain (lane 3, 619 bp and 1912 bp) and the recombinant (lane 5, 619 bp and 1081 bp) digested by *Sph*Ⅰ.


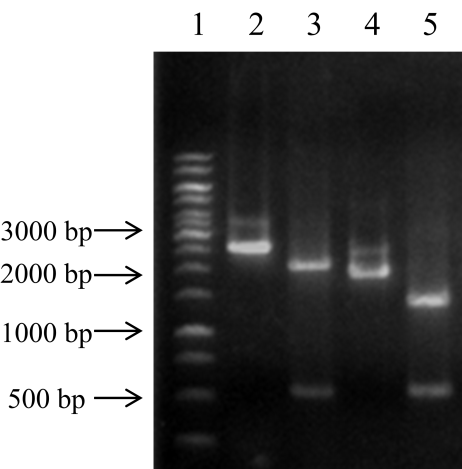


**Figure S3 The identification of the *pyrR* gene deletion.** Lane 1: marker. Lanes 2, 4: PCR products of the WT strain (lane 2, 2642 bp) and the recombinant (lane 4, 1904 bp) by using the primers pyrUP1 and pyrDN2. Lanes 3, 5: PCR products of the WT strain (lane 3, 531 bp and 2111 bp) and the recombinant (lane 5, 531 bp and 1373 bp) digested by *Pst*Ⅰ.


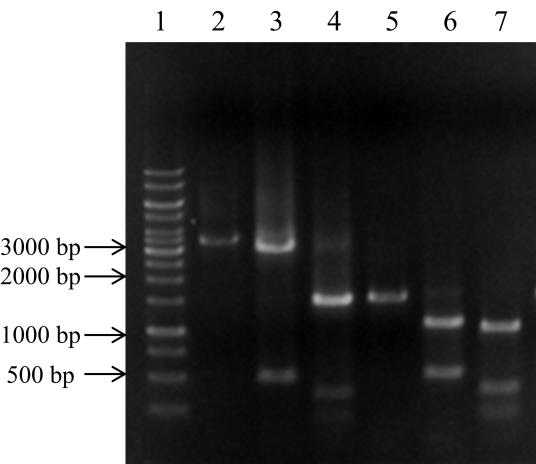


**Figure S3 The identification of the *nupC-pdp* gene deletion.** Lane 1: marker. Lanes 2, 5: PCR products of the WT strain (lane 2, 3433 bp) and the recombinant (lane 5, 1495 bp) by using the primers pnUP1 and pnDN2. Lanes 3, 6: PCR products of the WT strain (lane 3, 474 bp and 2959 bp) and the recombinant (lane 6, 474 bp and 1021 bp) digested by *Hin*dⅢ. Lanes 4, 7: PCR products of the WT strain (lane 4, 174 bp, 339 bp, 1452 bp and 1468 bp) and the recombinant (lane 7, 174 bp, 339 bp and 982 bp) digested by *Eco*RⅤ.

**Supplementary material 2**

**Deletion of the *cdd* gene.** The method of marker-free gene deletion was as described by Liu et al. [33, 34]. The fragment used for deleting the *cdd* gene was constructed as follows. The 0.9 kb *cat* (C) fragment was amplified from the pC194 plasmid using the primers Cat1c and Cat2c (Table 3). The 1.2 kb araR (R) fragment, including the whole coding region of the *araR* gene, was amplified from the *B. subtilis* 168 genome using the primers araR1qc and araR2E. The 1.2 kb UPcdd (U), 0.9 kb DNcdd (D) and 1 kb Gcdd (G) fragments were amplified from the *B. subtilis* 168 genome using the primers cddUP1 and cddUP2q, cddDN1 and cddDN2q, and cddG1E and cddG2, respectively. The U, D, C and R fragments were then ligated in the order U-D-C-R by splicing by overlapped extension PCR (SOE-PCR) using the primers cddUP1 and araR2E. Subsequently, the U-D-C-R and G fragments were digested by BamHI and ligated by T4 ligase and then were used to transform.

**Deletion of the *hom* gene.** The method of marker-free gene deletion was as described by Liu et al. [33, 34]. The fragment used for deleting the *hom* gene was constructed as follows. The 0.9 kb *cat* (C) fragment was amplified from the pC194 plasmid using the primers Cat1qm and Cat2m (Table 3). The 1.2 kb araR (R) fragment, including the whole coding region of the *araR* gene, was amplified from the *B. subtilis* 168 genome using the primers araR1qm and araR2m. The 1 kb UPhom (U), 0.7 kb DNhom (D) and 0.8 kb Ghom (G) fragments were amplified from the *B. subtilis* 168 genome using the primers homUP1 and homUP2, homDN1q and homDN2, and homG1q and homG2, respectively. These five PCR fragments were then ligated in the order U-D-C-R-G by splicing by overlapped extension PCR (SOE-PCR) using the primers homUP1 and homG2, and then were used to transform.

**Deletion of the *nupC-pdp* gene.** The method of marker-free gene deletion was as described by Liu et al. [33, 34]. The fragment used for deleting the *nupC-pdp* gene was constructed as follows. The 2.1 kb *cat*-araR (C-R) fragment was amplified from the U-D-C-R-G frgment of *hom* described before using the primers CR1qpn and CR2pn (Table 3). The 0.9 kb UPpn (U), 0.4 kb DNpn (D) and 1.3 kb Gpn (G) fragments were amplified from the *B. subtilis* 168 genome using the primers pnUP1 and pnUP2, pnDN1q and pnDN2, and pnG1q and pnG2, respectively. These four PCR fragments were then ligated in the order U-D-C-R-G by splicing by overlapped extension PCR (SOE-PCR) using the primers pnUP1 and pnG2, and then were used to transform.

**Overexpression of the *prs* gene**

To overexpress the *prs* gene, another *prs* gene copy controlled by P_AE_ expression cassette was integrated in chromosome the *xylR* gene locus (Figure 5). The method of marker-free gene modification was derived from Liu et al. [33, 34]. The specific method of gene modification was described as below. The 2.1 kb *cat*-araR (C-R) fragment was amplified from the U-D-C-R-G frgment of *hom* described before using the primers CR1qp and CR2p (Table 3). The 1.3 kb UPprs (U), 0.5 kb DNprs (D) and 0.8 kb Gprs (G) fragments were amplified from the *B. subtilis* 168 genome using the primers prsUP1 and prsUP2q, prsDN1q and prsDN2, and prsG1q and prsG2, respectively. The 0.9 kb prs (S) fragment, including the whole coding region of the *prs* gene, was amplified from the *B. subtilis* 168 genome using the primers prs1 and prs2. The 0.3 kb Promoter (P) fragment was whole sequence synthesized by AuGCT DNA-SYN Biotechnology Corporation (Beijing, China), including P_AE_ expression cassette, and ligated to plasmid pGH-A0981Gn. P fragment was amplified from plasmid pGH-A0981Gn by using the primers p1p and p2p. These six PCR fragments were then ligated in the order G-R-C-D-S-P-U by splicing by SOE-PCR using the primers prsUP1 and prsG2 and then be used to transform. DNA sequencing was done at AuGCT DNA-SYN Biotechnology Corporation (Beijing, China) by using primers CX1p and CX2p.

**Constitutive expression and overexpression of the *pyrG* gene**

To constitutively express the *pyrG* gene, 4 extra G residues were introduced at the 5′ ends of the *pyrG* ITR. To overexpress the *pyrG* gene, a [standardize](http://dict.youdao.com/w/standardize/)d promoter along with the the *gsiB* mRNA stabilizer (P_SB_ expression cassette) was inserted after the inherent *pyrG* promoter (Figure 6).The method of marker-free gene modification was derived from Liu et al. [33, 34]. The specific method of gene modification was described as below. Constitutively express the *pyrG* gene: the 0.9 kb *cat* (C) fragment was amplified from the pC194 plasmid using the primers Cat1g and Cat2g (Table 3). The 1.2 kb araR (R) fragment, including the whole coding region of the *araR* gene, was amplified from the *B. subtilis* 168 genome using the primers araR1qg and araR2g. The 1 kb UPpyrG (U), 1.1 kb DNpyrG (D) and 1.1 kb GpyrG (G) fragments were amplified from the *B. subtilis* 168 genome using the primers pyrGUP1 and pyrGUP2q, pyrGDN1 and pyrGDN2q, and pyrGG1q and pyrGG2, respectively. The 4 extra G residues were introduced by using the primer pyrGUP2q. These five PCR fragments were then ligated in the order G-R-C-D-U by splicing by SOE-PCR using the primers pyrGUP1 and pyrGG2 and then be used to transform. DNA sequencing was done at AuGCT DNA-SYN Biotechnology Corporation (Beijing, China) by using primers CX1g and CX2g. Overexpress the *pyrG* gene: The 1 kb UPpyrG2 (U2) fragment was amplified from the *B. subtilis* 168 genome using the primers pyrGUP1* and pyrGUP2q*. The 0.15 kb Promoter (P) fragment was whole sequence synthesized by AuGCT DNA-SYN Biotechnology Corporation (Beijing, China), including a [standardize](http://dict.youdao.com/w/standardize/)d promoter along with the the *gsiB* mRNA stabilizer (P_SB_ expression cassette), and ligated to plasmid pGH-A1285Gn. P fragment was amplified from plasmid pGH-A1285Gn by using the primers gsiB1 and gsiB2. The 4.3 kb DNpyrG-cat-araR-GpyrG (D-C-R-G) fragment was amplified from the U-D-C-R-G fragment in method 1 by using the primers pyrGDCRG1 and pyrGDCRG2. These three PCR fragments were then ligated in the order G-R-C-D-P-U by splicing by SOE-PCR using the primers pyrGUP1* and pyrGDCRG2 and then be used to transform. DNA sequencing was done at AuGCT DNA-SYN Biotechnology Corporation (Beijing, China) by using primers CX1p and CX2p.
